# Development of a High Stability Pd-Ni Alloy Thin-Film Coated SAW Device for Sensing Hydrogen

**DOI:** 10.3390/s19163560

**Published:** 2019-08-15

**Authors:** Wen Wang, Xueli Liu, Shengchao Mei, Mengwei Liu, Chao Lu, Minghui Lu

**Affiliations:** 1 Key Laboratory of Non-Destructive Testing Ministry of Education, Nanchang HangKong University, Nanchang 330063, China; 2 Institute of Acoustics, Chinese Academy of Sciences, Beijing 100190, China; 3 School of Electronic, Electrical and Communication Engineering, University of Chinese Academy of Sciences, Beijing 100049, China

**Keywords:** Pd-Ni alloy thin-film, hydrogen sensor, surface acoustic wave, working stability, differential oscillation loop

## Abstract

A Pd-Ni alloy thin-film coated surface acoustic wave (SAW) device is proposed for sensing hydrogen. The Pd-Ni thin-film was sputtered onto the SAW propagation path of a SAW device with a delay line pattern to build the chip-sized hydrogen sensor. The prepared sensor chip was characterized by employing a differential oscillation loop. The effect of the Pd-Ni film thickness on sensing performance was also evaluated, and optimal parameters were determined, allowing for fast response and high sensitivity. Excellent working stability (detection error of 3.7% in half a year), high sensitivity (21.3 kHz/%), and fast response (less than 10 s) were achieved from the 40 nm Pd-Ni alloy thin-film coated sensing device.

## 1. Introduction

As an efficient energy source, hydrogen gas features the advantages of clean energy, nontoxic, pollution-free, and high utilization ratio. However, the issue in hydrogen application that must remain the primary focus of attention is security, owing to the explosive nature of hydrogen in air. The early warning of hydrogen gas leakage is an essential way to improve the security, and it requires the hydrogen sensor to possess some essential properties, such as fast response, high sensitivity, and excellent working stability. Among the available approaches for sensing hydrogen, surface acoustic wave (SAW) devices feature some distinguishing characteristics such as chip size, fast response, low cost, satisfactory stability, and remarkable sensitivity [1,2,3,4,5]. Typical sensing devices are composed of a SAW device patterned by a resonator or delay line, and a sensitive thin film deposited on top of the SAW device. The adsorption in the sensitive thin film towards target gas molecules modulates the SAW propagation, and the corresponding shift in frequency is collected as the sensing signal. The SAW propagates along the piezoelectric substrate within one or two wavelengths, and the confined acoustic energy at the upper surface of the substrate is very sensitive to external loading, causing fast response and high sensitivity.

Many successes have been reported in the sensing of hydrogen gas by employing SAW technology over the past decades, since the pioneering work conducted by A. Amico [6]. Polymers, many metal oxides, and their bilayers have been used as the interface for sensing hydrogen [7,8,9,10,11,12,13,14,15], but the built sensors suffer from slow response, poor detection stability, or large power consumption because of the high working temperature required. Among various hydrogen-sensitive materials, palladium attracts substantial interest as the preferential candidate for sensing hydrogen owing to its high adsorption efficiency towards hydrogen molecules by a hydride (PdHx) formation at room temperature [16,17]. The induced so-called acoustoelectric coupling effect modulates the electricity distribution in the SAW field, and the corresponding shifts in acoustic velocity (frequency signal) can be collected for sensing the absorbed hydrogen gas [18]. However, the α–β phase transition occurring in the sensing process at hydrogen concentrations of 1%–2% degrades the stability and reproducibility of the sensing devices [16]. An effective way to cope with this is to dope moderate metals as alloy thin films. Some Pd alloys doped with metals such as Ag, Pt, Yi, and Cu have been used in hydrogen sensors [19,20,21,22,23,24]. Representative results were reported for Pd-Ni alloy thin-film coated SAW devices, with a very fast response time of 4 s and low detection limit of 3.7 ppm achieved [23,25]. However, the ball SAW devices suffer from the complex technique, and no further investigations on working stability benefiting from the alloy structure have been done in previous literatures. They are the main focus of this work.

In this contribution, a Pd-Ni thin film was coated onto the SAW propagation path to build the sensing device, and it was characterized by employing a differential oscillation loop. The effect of Pd-Ni thin-film thickness on the sensor response was investigated, and the optimal parameters were determined, offering fast response and high sensitivity. The typical sensor performance metrics, such as sensitivity, response speed, and working stability, were also evaluated experimentally.

## 2. Preparation of the Sensing Devices

A 160-MHz SAW delay line pattern with two aluminum (Al) transducers was fabricated photolithographically on 128° YX LiNbO_3_ wafer. The high electromechanical coupling coefficient (*K*^2^) of the piezoelectric wafer contributes well to the improvement the acoustoelectric sensitivity [18]. The distance between the two transducers was set to 3 mm to provide sufficient coating area. The strategic structures of single-phase unidirectional transducers (SPUDTs) and combed transducers were employed to form the SAW devices, offering lower insertion loss and excellent frequency stability [26]. Two transducers deposited on the wafer surface were composed of 180 and 60 electrode pairs. Corresponding electrode widths in SPUDTs were 6.6 μm (λ/4) and 3.3 μm (λ/8), respectively.

Then, a 50-nm SiO_2_ thin film was coated onto the transducers by employing PECVD (Plasma-Enhanced Chemical Vapor Deposition) to protect the electrodes in the process of Pd-Ni thin-film deposition. Next, the Pd alloy doped 10% Ni was sputtered onto the SiO_2_ surface between the two transducers by RF magnetron co-sputtering from Pd and Ni targets with a set ratio. Various Pd-Ni alloy film thicknesses were obtained by controlling the sputtering time, and the thickness was monitored by a step tester. Devices with various film thicknesses from 10 to 300 nm were prepared to evaluate the effect of thickness on gas adsorption. Additionally, for comparison, a 40-nm Pd thin film was prepared to demonstrate the long-term stability using a similar sputtering technique.

**Figure 1:**
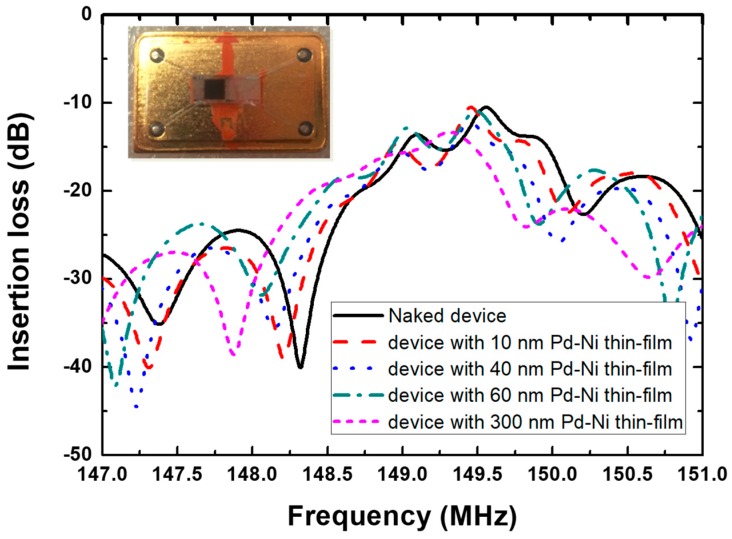
Measured frequency responses of the proposed sensing device depending on the Pd-Ni thin-film thickness, inset: picture of the prepared sensing device.

The prepared sensing device is pictured in the inset of Figure 1. The dark area denotes the deposited Pd-Ni alloy thin film. Using the network analyzer, the prepared sensing devices were characterized as shown in Figure 1. Obviously, with increases in Pd-Ni film thickness, the operation frequency of the devices decreased because of the mass loading effect. Additionally, notable increases in wave attenuation were observed with increases in Pd-Ni film thickness owing to the enhanced acoustoelectric coupling effect in the thicker films.

## 3. Sensor Experimental Setup

**Figure 2:**
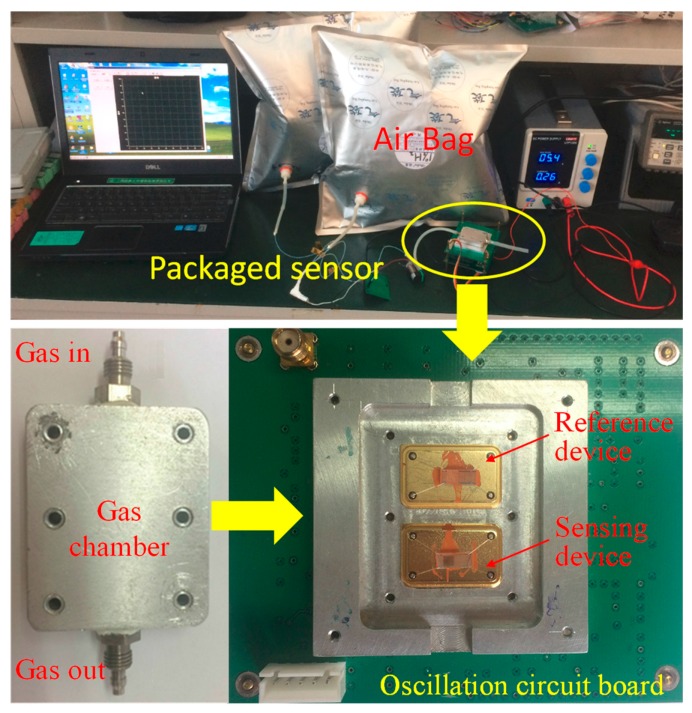
The experimental setup for characterizing the prepared sensing devices.

The prepared SAW sensing device was connected into a differential oscillation loop composed of an amplifier, phase shifter, mixer, and temperature control circuit to build the hydrogen sensor system. The differential oscillation frequency signal towards a reference device with no Pd-Ni thin film was collected using an FPGA (Field Programmable Gate Array)-based frequency acquisition module to evaluate the hydrogen gas to be detected. The sensing device and reference device were placed in a surface nickel-plated Al gas chamber with volume of 350 mL, as shown in Figure 2.

**Figure 3:**
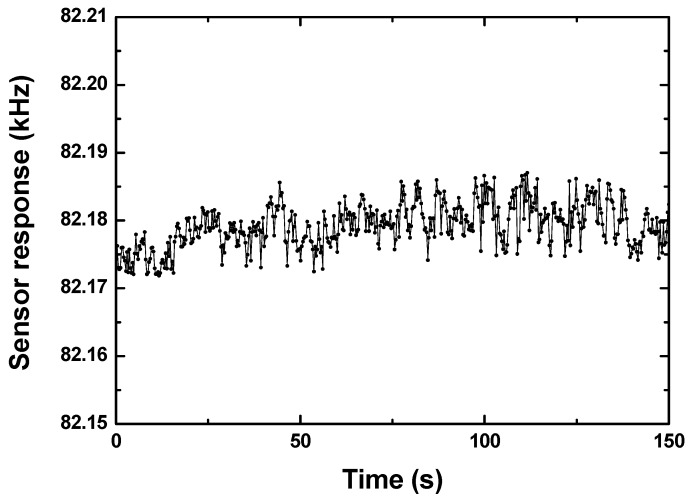
The measured baseline noise of the sensor system.

Then, the experimental setup depicted in Figure 2 including the developed SAW sensor system, solenoid valve for controlling the alternate sampling of the samples and N_2_, air pump with flow rate of 449 mL/min, power, air bag with hydrogen in nitrogen, and PC were set up to evaluate the prepared sensing devices. The baseline noise of the sensor system was also estimated by examining the frequency shift in a period of time, as shown in Figure 3. Less than ±5 Hz were observed in the measuring time.

## 4. Sensor Performance Evaluation

Using the experimental setup described in Figure 2, the prepared Pd-Ni alloy thin-film coated sensing devices were characterized, and corresponding sensor performance metrics such as sensitivity, repeatability, detection limit, and working stability were evaluated. Especially, the effect of Pd-Ni alloy thin-film thickness on sensor performance was conducted to extract the optimal parameters.

### 4.1. Repeatability

**Figure 4:**
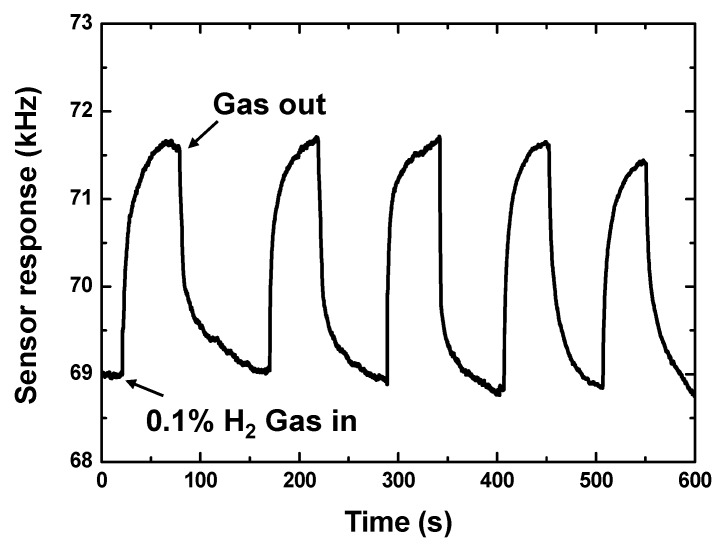
The repeatability measurement of the proposed sensing devices coated with 10 nm Pd-Ni alloy thin-film.

First, the repeatability of the 10 nm Pd-Ni alloy thin-film coated sensing device was evaluated by checking the sensor response from five consecutive 50 s on–off exposures to 0.1% hydrogen in pure N_2_ at room temperature (25 °C), as pictured in Figure 4. The obtained sensor response values from five reproducible runs were 2.61, 2.7, 2.7, 2.81, and 2.55 kHz, respectively. The corresponding standard deviation was calculated as 0.09, which indicates the perfect repeatability of the proposed sensing devices. Additionally, the response speed was evaluated as 15 s from the process of gas-in and gas-out, which was defined by the time to reach 90% of the stable sensor response value towards a given gas concentration. This indicates good repeatability with fast response, and a recovery time of ~15 s was achieved from the 10 nm Pd-Ni thin-film coated sensing device at room temperature.

### 4.2. The Effect of Pd-Ni Film Thickness on Sensor Performance

**Figure 5:**
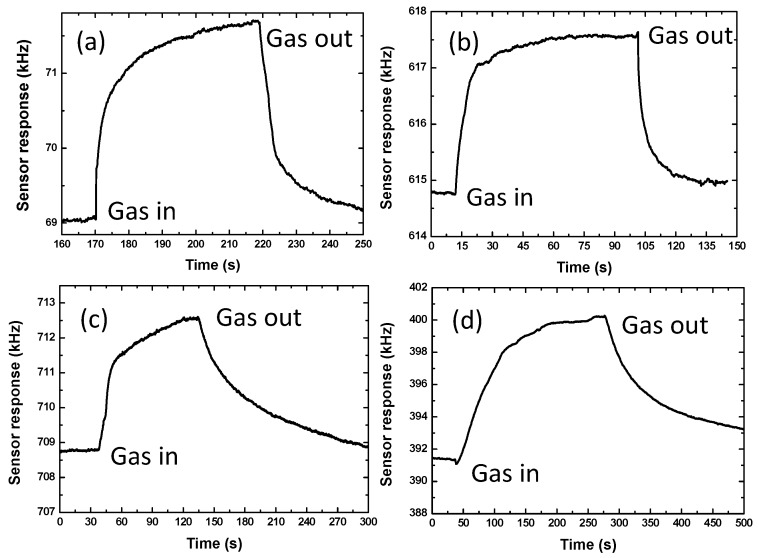
The measured sensor response towards 0.1% hydrogen gas employing the sensing devices coated with different Pd-Ni alloy thin-film thicknesses: (**a**) 10 nm; (**b**) 40 nm; (**c**) 60 nm; (**d**) 300 nm.

**Figure 6:**
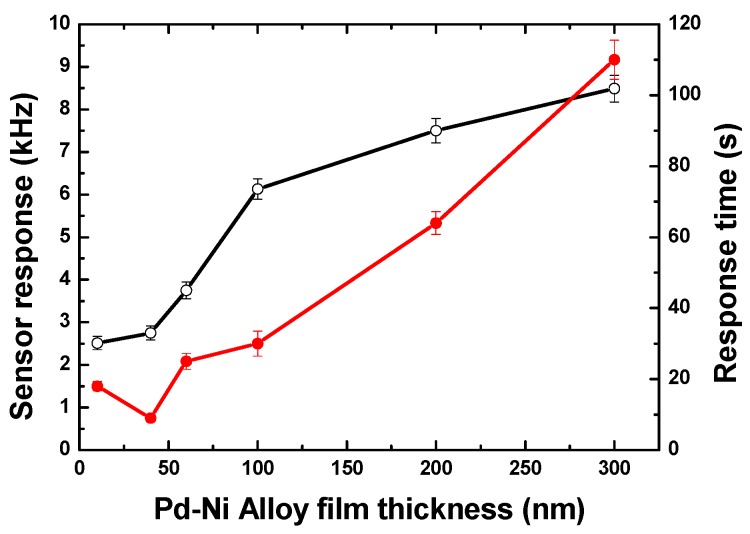
The relationship between the sensor response, response time, and the Pd-Ni thin-film thickness.

Because of the differences in gas adsorption behavior in Pd-Ni alloy thin-film with different thickness, the corresponding sensing performances were quite different. Figure 5 denotes the senor response towards 0.1% hydrogen from four sensing devices with different Pd-Ni film thicknesses, and the response values and response times depending on the film thickness are presented in detail in Figure 6. On one hand, the increases in Pd-Ni film thickness slow the diffusion velocity, leading to a slow response, but on the other, a thicker film adsorbs more gas molecules, and thus a higher sensitivity was expected. The highest sensor response of ~8.7 kHz was observed from the 300 nm Pd-Ni film coated device. Another interesting note was that the quickest response of ~7 s was obtained from the 40 nm film instead of the 10 nm one. The reason may be due to the poor thickness accuracy of the thin films less than 50 nm deposited by magnetic sputtering, in which the film thickness was monitored and controlled by the sputtering time. In conclusion, thin films could be a good choice when fast response is required. Moreover, higher sensitivity will be at the expense of response speed. In our design, a 40 nm Pd-Ni film was employed, in which a fast response (~7 s) and relatively high sensor response (2.75 kHz to 0.1% hydrogen) were obtained.

### 4.3. Sensitivity Evaluation

**Figure 7:**
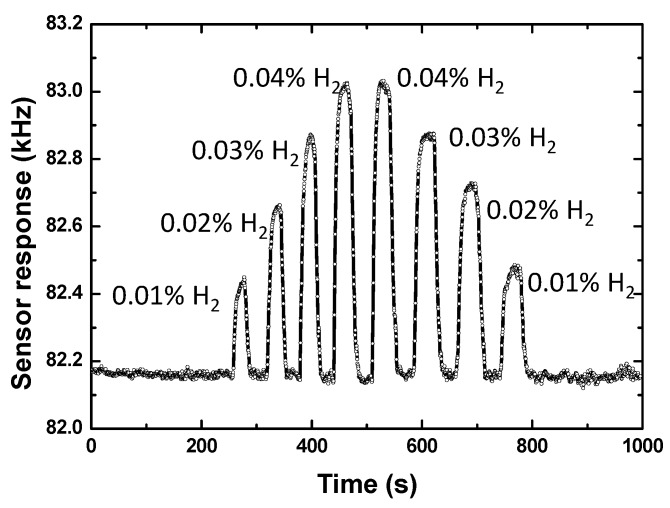
The measured sensor response relating to various hydrogen gas concentrations from the 40 nm Pd-Ni alloy thin-film coated sensing device.

**Figure 8:**
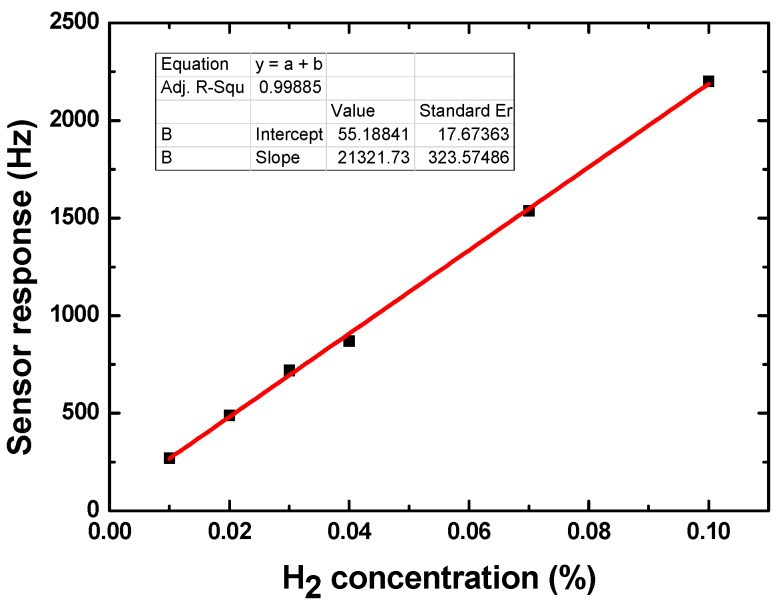
The sensitivity evaluation of the 40 Pd-Ni alloy thin-film coated sensing device.

The proposed 40 nm Pd-Ni alloy thin-film coated sensing device was exposed to different concentrations of hydrogen gas to evaluate its sensitivity. Figure 7 shows the sensor response towards various hydrogen gas concentrations (from 0.01% to 0.04%). With increases in hydrogen gas concentration, the sensor response amplitude increased accordingly. The sensor response values at various hydrogen gas concentrations are depicted in Figure 8, and the corresponding sensitivity was evaluated as 21.3 kHz/% (fitted by the least-squares method). As for another indicator, the detection limit can be obtained by theoretical prediction according to the relevant rules of IUPAC, that is, the detection limit is calculated as the lowest concentration of an analyte giving a sensor response of three times the baseline noise of the sensor itself. The observed sensor response from 0.01% hydrogen gas was ~270 Hz. Hence, a low detection limit of less than 0.001% is predicted because of the low baseline noise of ±5 Hz.

### 4.4. Long-Term Stability

**Figure 9:**
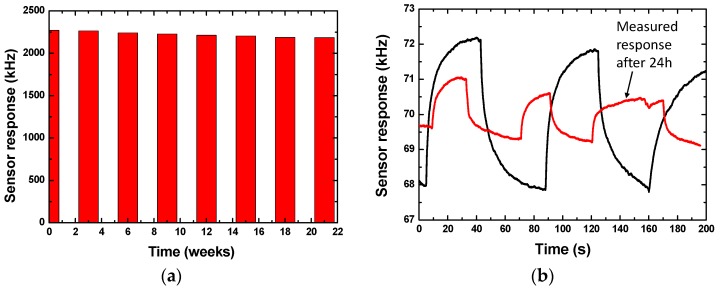
The measurement of the long-term stability of the 40 nm Pd-Ni alloy thin-film coated device (**a**), and the 40 nm Pd thin-film coated device (**b**).

In a long-run service, the gas sensor will suffer from the time drift, thereby weakening the long-term working stability. In this work, the sensor response from 40 nm Pd-Ni alloy thin-film coated sensing devices towards 0.1% hydrogen gas at 25 °C was collected and recorded intermittently over half a year, as depicted in Figure 9a. Usually, the working stability can be represented by detection error (e) defined by e = (X − X_0_)/X_0_ × 100%, where X and X_0_ denote the sampling value and initial value, respectively. The half-year detection error was ~3.7%—much better than that from the 40 nm Pd thin-film coated device described in Figure 9b. A larger detection error over 61.9% was observed from the Pd film coated device in only two days, which means the Ni doping in Pd suppressed the lattice destruction in gas adsorption, and significantly improved the long-term stability.

### 4.5. Selectivity Testing

**Figure 10:**
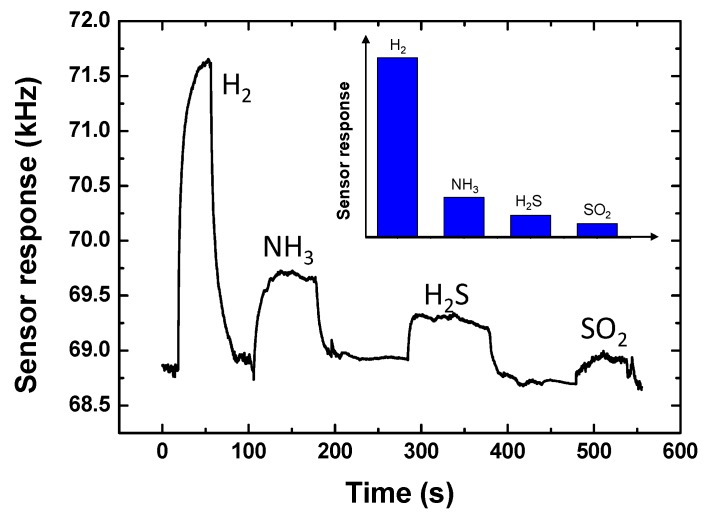
The selectivity testing of the proposed Pd-Ni alloy thin-film coated sensing device.

An experiment was performed to describe the selectivity of the proposed 40 nm Pd-Ni alloy thin-film coated sensing device by exposing the developed sensor to various common interfering gases such as H_2_S, SO_2_, and NH_3_. Figure 10 gives the response profiles of the sensing device towards 0.1% hydrogen, 100 ppm H_2_S, 100 ppm SO_2_, and 100 ppm NH_3_. Obviously, the sensor response towards hydrogen was larger than that to the interfering gases, which indicates the proposed SAW sensing device features excellent selectivity.

## 5. Conclusions

In this work, a Pd-Ni alloy thin-film coated SAW devices was constructed to build a hydrogen gas sensor, and was characterized by employing a differential oscillation loop. The effect of the Pd-Ni thin-film on the sensor response was investigated experimentally, and the optimal film thickness was determined. Fast response (~7 s), high sensitivity (21.3 kHz/%), low detection limit (0.001%), and excellent long-term stability (3.7%) were achieved successfully at room temperature.
